# The NS Segment of H1N1pdm09 Enhances H5N1 Pathogenicity in a Mouse Model of Influenza Virus Infections

**DOI:** 10.3390/v10090504

**Published:** 2018-09-17

**Authors:** Olivier Ferraris, Jean-Sébastien Casalegno, Emilie Frobert, Maude Bouscambert Duchamp, Martine Valette, Frédéric Jacquot, Hervé Raoul, Bruno Lina, Michèle Ottmann

**Affiliations:** 1 Laboratoire de Virologie et Pathologies Humaines Virpath, CIRI, Centre International de Recherche en Infectiologie, Université de Lyon, Inserm U1111, CNRS UMR5308, École Normale Supérieure de Lyon, Université Claude Bernard Lyon 1, 69372 CEDEX 08 Lyon, France; olivier.ferraris@yahoo.fr (O.F.); jean-sebastien.casalegno@chu-lyon.fr (J.-S.C.); emilie.frobert@chu-lyon.fr (E.F.); bruno.lina@univ-lyon1.fr (B.L.); 2 Laboratoire de Virologie, Institut des Agents Infectieux, Groupement Hospitalier Nord des Hospices Civils de Lyon, 69317 CEDEX 04 Lyon, France; maude.bouscambert-duchamp@chu-lyon.fr (M.B.D.); martine.valette@chu-lyon.fr (M.V.); 3 Laboratoire de Virologie, Centre National de Référence Virus des Infections Respiratoires, Groupement Hospitalier Nord des Hospices Civils de Lyon, 69317 CEDEX 04 Lyon, France; 4 Laboratoire P4 Jean Mérieux Inserm US003, 69365 CEDEX 07 Lyon, France; frederic.jacquot@inserm.fr (F.J.); herve.raoul@inserm.fr (H.R.)

**Keywords:** virus, influenza, influenza A, H5N1, H1N1pdm, reassortment, pathogenicity, mouse model

## Abstract

In 2009, the co-circulation of H5N1 and H1N1pdm09 raised concerns that a reassortment event may lead to highly pathogenic influenza strains. H1N1pdm09 and H5N1 are able to infect the same target cells of the lower respiratory tract. To investigate the capacity of the emergence of reassortant viruses, we characterized viruses obtained from the co-infection of cells with H5N1 (A/Turkey/13/2006) and H1N1pdm09 (A/Lyon/969/2009 H1N1). In our analysis, all the screened reassortants possessed the PB2, HA, and NP segments from H5N1 and acquired one or two of the H1N1pdm09 segments. Moreover, the in vivo infections showed that the acquisition of the NS segment from H1N1pdm09 increased the virulence of H5N1 in mice. We conclude, therefore, that reassortment can occur between these two viruses, even if this process has never been detected in nature.

## 1. Introduction

Since 1997, infections due to A/H5N1 avian influenza have been reported, with a high fatality rate amongst the confirmed cases [1]. Despite this continuing threat, these viruses have not been able to engage in a sustained human-to-human transmission. The molecular adaptations that would result from this transmission have been determined through controlled experiments in a ferret model, but the circulating viruses have not acquired this capacity thus far [2,3]. However, the emergence of such adapted viruses remains a major public health concern, justifying the enhanced surveillance in cases of detection of clusters of H5N1 viruses in poultry or wild birds. As observed in the last three influenza pandemics, genetic reassortment has been a major factor in the introduction of new virus subtypes in humans [4,5]. However, this reassortment needs to be combined with the adaptation of the receptor binding site (RBS) of the HA segment of the zoonotic virus to the human α2,6-linked sialic acid. As of today, A/H5N1 viruses from all the clades and subclades have demonstrated a high affinity to sialic acid linked to galactose by α2,3-linkages and have been shown to bind poorly to the human receptor. However, since 2009 H1N1pdm09 viruses continue to circulate in humans as a recurrent seasonal influenza virus and those viruses may have a dual tropism [6,7]. Thus, human co-infections by both H5N1 and H1N1 could be possible. The HA segment from H5N1 is a virulence factor due to the presence of multiple basic amino acids required for efficient HA cleavage by ubiquitous intracellular furin-like proteases.

Based on a method already in use, we generated H5N1-H1N1 reassortant viruses by co-infection in vitro. Then, we evaluated the replication in vitro and the virulence in a mouse model. We report that the HA, PB2, and NP segments of H5N1 are present in all the generated reassortant viruses in our experimental conditions.

## 2. Materials and Methods 

### 2.1. Ethics Statement

The animal care and experimental procedures were approved by the Committee on the Animal Experiments CECCAPP (CE015) of the SFR Biosciences (Permit numbers: P4 2009 003, P4 2009 004, P4 2010 001, P4 2010 002, P4 2011 001).

### 2.2. Laboratory Facilities, Biosafety, and Biosecurity

All the experiments using parental H5N1 and reassortant H5N1 viruses containing H1N1 segments were conducted in the BSL4 containment laboratory “Laboratoire P4 Jean Mérieux” in Lyon. The biosafety protocols were approved by the BSL4 “P4 Jean Mérieux” Biosafety Committee. The RNA was isolated using techniques documented to inactivate the virus particles in the samples before their removal from the BSL4 laboratory. All the experimental studies with H5N1 viruses were performed before the moratorium.

### 2.3. Cells and Viruses

Madin–Darby canine kidney (MDCK) cells were maintained in UltraMDCK serum-free medium (Lonza, Basel, Switzerland) supplemented with 2 mM l-glutamine, 200 IU/mL penicillin (Lonza), and 200 IU/mL Streptomycin (Lonza) at 37 °C and 5% CO_2_. A/Lyon/969/2009 H1N1 (H1N1pdm09) was the first H1N1 pandemic influenza virus isolated by CNR France-Sud in 2009. A/Turkey/13/2006 H5N1 (H5TK13) is a clade 2.2, highly pathogenic influenza virus [8]. The H1N1pdm09 and TK13 viruses were propagated in the MDCK cells at 34 °C in Eagle’s minimum essential medium (EMEM Lonza) supplemented with 1 µg/mL TPCK-treated trypsin (Sigma-Aldrich, St. Louis, MO, USA). The infectious viral titers were determined by 50% tissue culture infectious dose (TCID_50_) analysis in the MDCK cells from 4 replicates by means of endpoint titration in the MDCK cells using the Reed and Muench method.

### 2.4. In Vitro Co-Infections

The co-infections were performed as previously described [9]. Briefly, the MDCK cells were first infected with H1N1pdm09 at a multiplicity of infection (MOI) of 0.5 for 1 h at 34 °C. The cells were washed and incubated for 3 additional h at 34 °C in EMEM containing 1 µM oseltamivir (kindly provided by Hoffmann-La Roche, Ltd., Basel, Switzerland). The cells were then washed and infected with H5TK13 at a MOI of 0.5 for 1 h at 34 °C. The cells were finally washed and cultured for 18 h at 34 °C in EMEM containing 1 µM oseltamivir. The progeny viruses were then plaque purified: 10-fold serial dilutions of supernatants were used to inoculate the MDCK cells for 1 h at 34 °C, and then, the cells were overlaid with EMEM containing 0.55% agar. After 48 h at 34 °C, 50 viral clones were isolated, propagated in the MDCK cells, and stored at −80 °C. The genotyping was performed by Sanger sequencing of the cDNA amplified with sets of primers identifying the gene origins [10,11].

### 2.5. Replication Kinetics

The replication curves were generated by inoculating the MDCK cells at a MOI of 0.0001 TCID_50_ per cell. The supernatants were sampled at 18, 24, 42, 48, and 66 h after inoculation and stored at −80 °C titered, as described above.

### 2.6. Animal Experiments

First, 6- to 8-week-old female BALB/c mice (Charles River Laboratories, Wilmington, MA, USA) were anesthetized with isoflurane and intranasally inoculated with PBS or PBS-diluted viruses (10^2^–10^6^ TCID_50_ of each virus) in 20 µL PBS. The mice were weighed and monitored daily for clinical signs of infection. Animals with prostration signs or with severe weight loss (≥25%) were euthanized, and their tissues were harvested and frozen at −80 °C. The thawed tissues from the 10^5^ TCID_50_-infected mice were weighed and homogenized in 0.5 mL PBS using a Tissuelyser II (Qiagen, Hilden, Germany), and the viral titers were determined by TCID_50_ analysis. The viral RNAs from sera were extracted using a QIAmp viral RNA mini kit (Qiagen). Real-time RT-PCR was performed with 5 µL of the sample using a SuperScript III Platinum one-step qRT-PCR system (Invitrogen, Carlsbad, CA, USA) in Mastercycler Realplex 2 (Eppendorf, Hamburg, Germany) with appropriate primers for the detection of influenza A and M segment [12].

### 2.7. Statistical Analyses

The statistical analyses were done with XLSTAT life 2012.5 software (Addinsoft, Paris, France). Student’s *t*-tests were used to compare survival and virus titers in organs. Kaplan–Meier analysis was used to estimate the mean survival time.

## 3. Results

### 3.1. Genetic Characterization of Reassortant Viruses

The reassortant viruses were generated by H5TK13 virus superinfection of the MDCK cells previously infected by H1N1pdm09. The use of oseltamivir between the first and second round of infection reduced the NA activity at the surface of the infected cells in order to allow virus superinfection [9]. 

Most of the 50 randomly screened clones derived entirely from the parental H5N1 TK13 virus except six H5N1-H1N1pdm09 reassortant viruses containing one or two of the segments from the H1N1pdm09 virus (Table 1). All the H5N1-H1N1pdm09 reassortant viruses contained the PB2, HA, and NP segments from H5TK13, and three of them incorporated one H1N1pdm09 segment—H5TK13 PB1, H5TK13 M, and H5TK13 NS1—whereas three reassortant viruses incorporated two H1N1pdm09 segments: H5TK13 M NA, H5TK13 PA NS, and H5TK13 NA NS.

### 3.2. In Vitro Characterization of Reassortant Viruses

**Figure 1:**
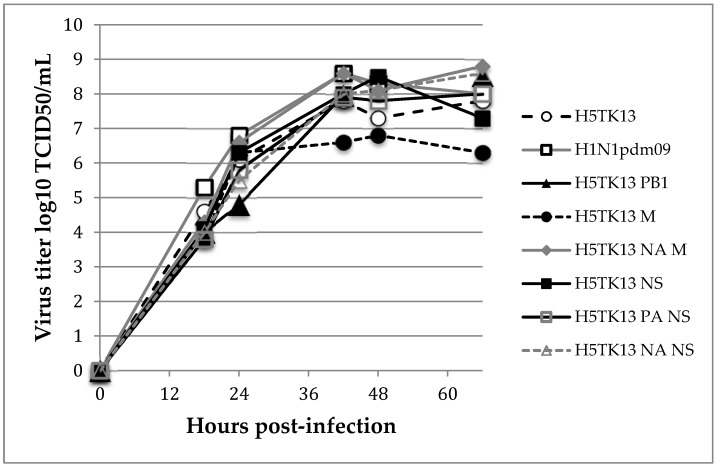
Replication of the wild-type and reassortant H5N1-H1N1pdm09 viruses in the MDCK cells. The MDCK cells were infected with the wild-type H5TK13 virus or the reassortant derived from the H5TK13 viruses at an MOI of 0.0001. The supernatants were titrated in the MDCK cells.

The growth characteristics of the H5N1 reassortant viruses were determined by the replication kinetics in the MDCK cells. The MDCK cells were inoculated with the reassortant viruses at a MOI of 0.0001, and the supernatants were harvested at the indicated times. The virus titration in the supernatants was performed in the MDCK cells in quadruplicate. As shown in Figure 1, the maximal viral production of the wild-type H5TK13, H1N1pdm09, and H5N1-H1N1pdm09 reassortant viruses was reached at 42 h and ranged between 10^7.8^ and 10^8.8^ TCID_50_/mL at 66 h post-infection, except for the H5TK13 M reassortant virus. The replication curve of the H5TK13 M reassortant bend at 24 h post-infection was 1.5 logs lower than that of the wild-type H5TK13.

### 3.3. Pathogenicity of Reassortant Viruses in Mice

To investigate the pathogenicity of the reassortant viruses, groups of six mice were infected intranasally with 10-fold dilution from 10^2^ to 10^6^ TCID_50_ of each virus. The mean mouse lethal dose 50% (MLD_50_) showed that the H5TK13 NS virus displayed a lower MLD_50_ than the wild type (10^0.3^ and 10^1^, respectively; Table 2). The incorporation of a second H1N1pdm09 segment reduced the MLD_50_, as demonstrated, for H5TK13 PA NS and H5TK13 NA NS (10^1.8^ and 10^3.7^, respectively, versus 10^0.3^ for H5TK13 NS). The other reassortant viruses had higher MLDs_50_ than did H5TK13, up to 100 times for H5TK13 PB1 and up to 1000 times for H5TK13 M and H5TK13 NA M (10^7.7^ and 10^8.8^, respectively).

**Figure 2:**
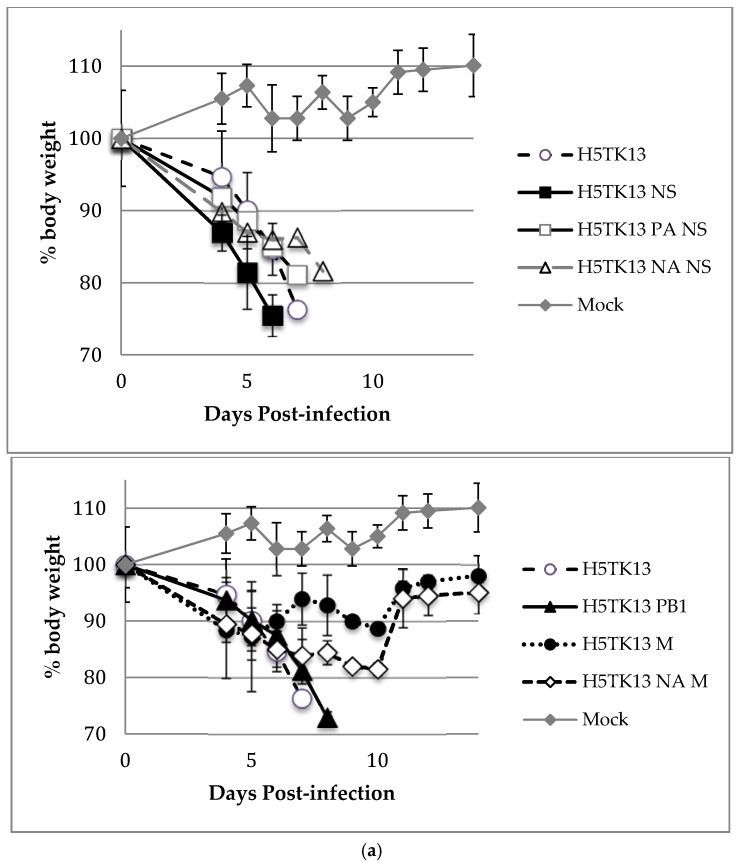

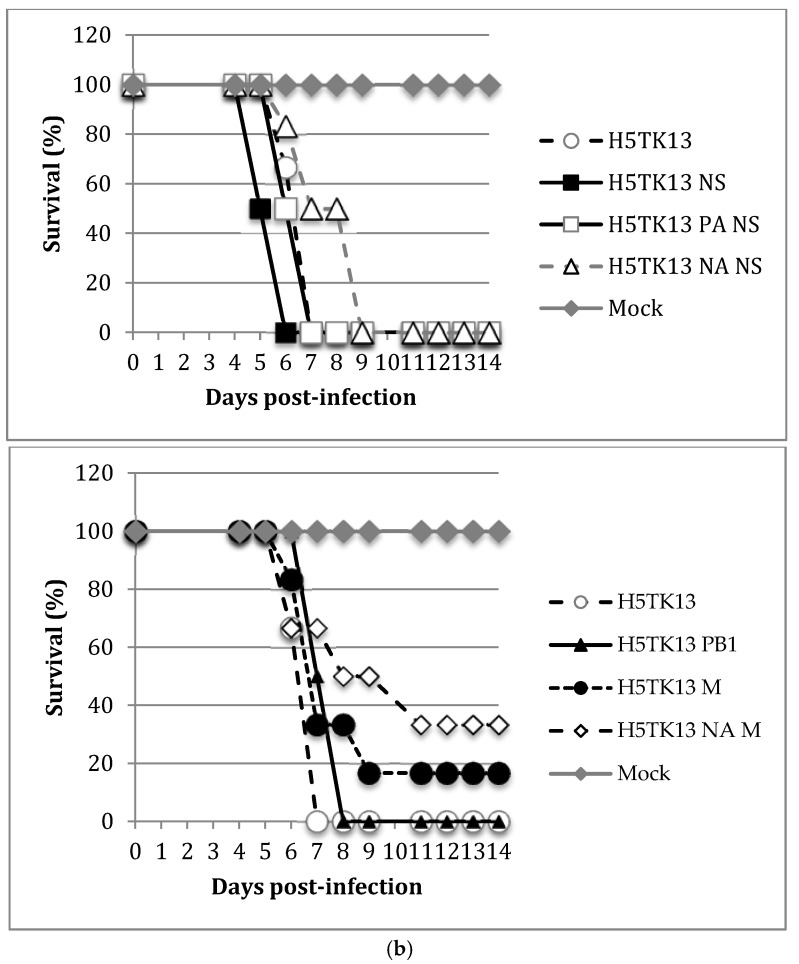
Pathogenicity of reassortant viruses in mice. Morbidity (weight loss) (**a**) and mortality (**b**) of mice inoculated with 10^5^ TCID_50_ reassortant viruses. The data for the mock and H5TK13-infected mice are reported in each graph.

The mean survival time was calculated for the 10^5^ TCID_50_ virus dose (Table 2). The results are significantly different (*p* < 0.001) compared with the H5TK13 wild type, except for H5TK13 M and H5TK13 PA NS. The virus H5TK13 NS was the most virulent, with the lowest mean survival time after inoculation (5.5 days versus 6.5 days for the wild-type H5TK13), also shown in Figure 2 by the mouse body loss and mortality. The results of the weight loss curves and mortality are consistent with MLD_50_ (Figure 2).

Given that virulence is also dependent on the spread of the virus replication, we determined the viral load in the organs of the mice inoculated with a 10^5^ TCID_50_ virus dose and euthanized at the onset of symptoms and weight loss (Table 2). While the virus titer in the lungs was not statistically different from H5TK13, it should be noted that all the reassortants were restricted to the respiratory system except for H5TK13 NS, which was detected in the spleen and brain as the H5TK13 wild type. The presence of the virus in sera was evaluated by RT-PCR and did not reveal any significant difference (*p* < 0.05) between the reassortant viruses and the H5TK13 parental strain, even for the H5TK13 NS reassortant.

## 4. Discussion

Genetic reassortment is a common event with the influenza A virus, and it can generate a pandemic virus as with the H1N1pdm09 in 2009. The acquisition of a human-transmissible phenotype by the H5N1 avian influenza viruses through reassortment with the human influenza viruses represents a major pandemic threat. Hence, studies of the reassortant viruses are useful for identifying genetic patterns involved in the pathogenicity and/or transmissibility of such viruses. In mice, H5N1 virulence factors have been identified in PB1 (PB1-F2), PB2, HA, and NS (NS1) [13]. The reassortant viruses obtained in ferrets with a H5N1 background and a human H3N2 segment [14] or recombinant viruses obtained by reverse genetics [15,16] have harbored equal or less pathogenicity than the parental H5N1 virus, except for those that acquired the H3N2 PB2 segment, which showed an enhanced virulence [16]. Similarly, the introduction of the HA segment of H5N1 in a H1N1pdm09 genetic background enhanced the virulence of the reassortant viruses in mice as compared with the parental virus H1N1pdm09 [17]. Finally, specific mutations in the HA segment of the H5N1 virus rendered the virus transmissible between ferrets [2,3]. 

However, the introduction of other gene segments may decrease the pathogenicity. For example, we show here that the presence of the PB1 segment of human origin [18,19] in a H5N1 background decreased its pathogenicity. This is consistent with other studies [15,16]. The combination of H1N1pdm09 PB1 with PB2, PA, and NP from H5N1 is probably not optimal in the polymerase complex [20,21] for the synthesis of the viral RNA and makes it a less pathogenic virus than the parental ones.

While reverse genetics systems force reassortment, we used co-infection to identify segments that may spontaneously be packaged in progeny viruses. In this study, we showed that the H5TK13 M reassortant replicates less efficiently in the MDCK cells (Figure 1) and is less pathogenic than the parental H5N1 strain. The M segment encodes the matrix protein (M1) and the ion channel (M2). The M1 protein plays a key role in viral morphogenesis by interacting with the envelope glycoproteins HA and NA and with the vRNPs [22,23]. Although the H1N1pdm09 M segment has been incorporated into the H5N1 background (H5TK13 M), the H1N1pdm09 M1 protein does not allow for the optimum interactions between the H5N1 viral proteins and causes a loss of infectivity and virulence, slightly accentuated by the additional incorporation of the H1N1pdm09 NA segment. Moreover, the acquisition of the H1N1pdm M segment could contribute to promoting the transmissibility, as described in a guinea pig model [24].

The incorporation of a heterologous NA from a seasonal or pandemic H1N1 strain similarly weakened the H5TK13 reassortant viruses, probably due to an imbalance between the H1N1pdm09 NA activity and the H5N1 HA affinity [10].

The H5TK13 NS reassortant virus contains the H1N1pdm09 NS segment encoding NS1 and NEP in the H5N1 genetic background. The H1N1pdm09 NS segment is a porcine, derived from the 1918 pandemic virus. The adaptation of the H1N1 virus in swine led to multiple mutations in the NS1 protein, the K217E substitution that abolishes the NS1 binding to the cellular protein Crk/CrkL (the adapter protein family involved in signal transduction), and the deletion of the 11 C-terminal residues. The C-terminus of the entire NS1 protein contains a binding domain to PABPII, a protein involved in the inhibition of the polyadenylation of the cellular pre-mRNA, and a binding PDZ domain RSEV with a motif present in human H1N1 [25].

The reintroduction of the C-terminal of NS1 into the H1N1pdm09 virus had no effect on the virus replication in the human cells or the swine model [26]. Thus, the virus H1N1pdm09 is optimized to replicate efficiently without involving some of the functions of NS1 that have been identified in other influenza A viruses. This may explain its worldwide dissemination and low pathogenicity. However, H5TK13 NS is slightly more virulent in mice than the H5N1 parental virus. Studies have reported that reassortant viruses H5N1xH3N2 (the H3N2 NS segment in the H5N1 genetic background) have showed a lower virulence than the H5N1 parental virus [16]. Note that the NS1 protein of the H3N2 virus is not truncated as is the H1N1pdm09 NS1 protein.

While the NS1 protein of H1N1pdm09 less efficiently stimulates the translation of a reporter gene in vitro and inhibits less polyadenylation of the cellular pre-mRNA in A549 cells compared with that of its avian counterparts due to the lack of binding to the cell factor IIAP, viruses with a truncated NS1 are more virulent in mice [27], rendering our results consistent with those data. The addition of a second segment (NA or PA) from the H1N1pdm virus in the genetic background of H5N1 (H5TK13 NA NS and H5TK13 PA NS) results in less virulence than H5TK13NS. Two hypotheses support this reduced virulence: (1) An unfavorable H5N1 HA/H1N1pdm NA balance for the H5TK13 NA NS virus and (2) a reduction in the efficiency of the replication/transcription of the polymerase complex for the H5TK13 PA NS virus.

## 5. Conclusions

In conclusion, the reassortant viruses obtained by co-infection [28], our results, and the recombinant viruses obtained by reverse genetics [16] showed that H5N1 and H1N1pdm09 had high genetic compatibility. Nevertheless, no reassortant has been detected today in humans, either because the reassortment did not occur or because the infection has been inapparent and not transmitted.

## Figures and Tables

**Table 1:** Genetic composition of the viruses obtained from the co-infection of the Madin–Darby canine kidney (MDCK) cells with the H1N1 and H5N1 viruses (H1N1 segments in bold).

	1	2	3	4	5	6	7	8
Reassortant Virus	PB2	PB1	PA	HA	NP	NA	M	NS
H5TK13	H5	H5	H5	H5	H5	H5	H5	H5
H5TK13 PB1	H5	**H1**	H5	H5	H5	H5	H5	H5
H5TK13 M	H5	H5	H5	H5	H5	H5	**H1**	H5
H5TK13 M NA	H5	H5	H5	H5	H5	**H1**	**H1**	H5
H5TK13 NS	H5	H5	H5	H5	H5	H5	H5	**H1**
H5TK13 PA NS	H5	H5	**H1**	H5	H5	H5	H5	**H1**
H5TK13 NA NS	H5	H5	H5	H5	H5	**H1**	H5	**H1**

**Table 2:** Virulence of the H5N1/H1N1pdm09 reassortant viruses in mice.

Virus and Reassortants	log10 MLD_50_^†^	Mean Survival Time (Days) ^‡^	Virus Titer ^§^ (log10 TCID_50_/gram ± SD) (No. Positive Mice/Total No. of Mice)			Copies × 10^5^/mL ± SD (No. of Positive Mice/Total No. of Mice)
			Lung (6/6)	Spleen ^#^	Brain ^#^	Serum No Significant Difference *p* < 0.05
H5TK13 WT	1.0	6.5	6.1 ± 0.3	2.5 (1/6)	4.9 ± 0.6 (5/6)	7.0 ± 1.3 (6/6)
H5TK13 PB1	2.3	7.5	5.6 ± 0.3	<(0/6)	<(0/6)	7.8 ± 0.5 (4/6)
H5TK13 M	4.4	7.7	6.4 ± 0.4	<(0/6)	<(0/6)	7.2 ± 1.1 (6/6)
H5TK13 NA M	4.6	8.8	6.4 ± 0.1	<(0/6)	<(0/6)	6.4 ± 0,8 (6/6)
H5TK13 NS	0.3	5.5	6.6 ± 0.3	3.2 ± 0.3 (3/6)	3.0 ± 0.6 (3/6)	5.9 ± 0.2 (4/6)
H5TK13 PA NS	1.8	6.5	6.5 ± 0.1	<(0/6)	<(0/6)	8.7 ± 0.9 (6/6)
H5TK13 NA NS	3.7	8.3	6.8 ± 0.6	<(0/6)	<(0/6)	6.8 ± 0.7 (6/6)

^†^ The mean mouse lethal dose 50% (MLD_50_) was determined by the Reed and Muench method; ^‡^ The mean survival time was calculated for the 10^5^ 50% tissue culture infectious dose (TCID_50_) by Kaplan–Meier analysis; ^§^ Virus titer in the organs was measured from the 10^5^ TCID_50_-infected mice; ^#^ <: the titer was below the detection limit (0.5 TCID_50_/mL).
